# Effects of Grape Extract Supplementation on Postpartum Reproductive Responses in Beef Cows: A Pilot Study

**DOI:** 10.3390/ani16121779

**Published:** 2026-06-09

**Authors:** Inga Merkelytė, Algirdas Urbšys, Rasa Nainienė, Audronė Rekešiūtė, Artūras Šiukščius

**Affiliations:** 1 Department of Animal Breeding and Reproduction, Animal Science Institute, Lithuanian University of Health Sciences, R. Zebenkos 12, LT-82317 Baisogala, Lithuania; algirdas.urbsys@lsmu.lt (A.U.); rasa.nainiene@lsmu.lt (R.N.); arturas.siukscius@lsmu.lt (A.Š.); 2 Animal Reproduction Laboratory, Faculty of Veterinary Medicine, Veterinary Academy, Lithuanian University of Health Sciences, Tilžes Str. 18, LT-47181 Kaunas, Lithuania; audrone.rekesiute@lsmu.lt

**Keywords:** infrared thermography, estrus detection, beef cattle, postpartum period, reticulorumen temperature, artificial intelligence, reproductive performance, grape extract, polyphenols, precision livestock farming

## Abstract

The postpartum period in beef cows is characterized by complex physiological and reproductive changes that may influence the postpartum reproductive changes and estrus expression. This exploratory pilot study evaluated postpartum reproductive responses in Angus cows receiving grape extract supplementation while investigating associations among thermographic, behavioral, hormonal, and reticulorumen temperature indicators associated with estrus expression. Nineteen Angus cows were assigned to a control group (C; *n* = 10) or a treatment group receiving slow-release grape extract boluses (T; *n* = 9). From calving until artificial insemination, ocular and vulvar thermographic images, blood samples, and physiological measurements were obtained weekly between 10:00 and 11:00 a.m. Reticulorumen temperature and activity data were continuously recorded using intraruminal sensors. Kaplan–Meier survival analysis demonstrated an earlier onset of postpartum estrus in supplemented cows compared with controls (*p* = 0.010). Mean time to first estrus was 23.88 ± 1.86 days in the T group and 39.82 ± 5.05 days in the C group. No significant differences were observed between groups for most individual physiological or hormonal variables. Exploratory correlation analysis revealed moderate associations among vulvar temperature, ocular temperature, activity, estrus index, and reticulorumen temperature indicators. However, because diagnostic accuracy was not evaluated, these findings should not be interpreted as validation of estrus detection performance. The results suggest that multimodal physiological monitoring may provide complementary information related to postpartum estrus expression in beef cows, while grape extract supplementation may be associated with earlier postpartum reproductive recovery. Due to the exploratory study design and limited sample size, further studies are required to validate these preliminary observations.

## Simple Summary

Early detection of estrus after calving is important for maintaining reproductive efficiency in beef cattle; however, postpartum estrus may be difficult to identify because physiological and behavioral signs are often subtle. This exploratory pilot study evaluated postpartum reproductive responses in Angus cows receiving grape extract supplementation and investigated associations among infrared thermography, reticulorumen temperature, activity monitoring, and hormonal indicators related to estrus expression. Cows supplemented with grape extract returned to estrus earlier postpartum compared with non-supplemented cows. In addition, moderate associations were observed among thermal, behavioral, and physiological indicators. Although the study was not designed to validate estrus detection accuracy, the findings suggest that multimodal physiological monitoring may provide complementary information related to estrus expression in beef cows. Further studies involving larger populations are required to confirm these preliminary observations.

## 1. Introduction

Efficient reproductive management remains one of the most critical factors determining productivity and profitability in beef cattle systems. Early detection of estrus and timely insemination are essential for maintaining optimal calving intervals and improving herd reproductive performance. However, estrus expression in postpartum cows may be less evident and influenced by factors such as hormonal fluctuations, negative energy balance, postpartum anestrus, housing conditions, environmental temperature, and limitations associated with visual observation, making accurate identification challenging [1,2].

Traditionally, estrus detection has relied on behavioral observations, such as increased activity and mounting behavior. While these indicators are widely used, their reliability may be limited due to variability among animals, postpartum physiological status, environmental conditions, and limitations inherent to human-based observations [1]. Consequently, there has been growing interest in integrating physiological and technological approaches into reproductive monitoring systems. Among these, automated activity monitoring and artificial intelligence (AI)-assisted systems have been increasingly investigated as supportive tools for monitoring behavioral and physiological changes associated with estrus [3,4]. However, the practical application of these technologies in beef cattle systems may be influenced by extensive or semi-extensive management conditions, including larger grazing areas and limited internet or network connectivity in remote farming environments.

In recent years, non-invasive physiological measurements, particularly infrared thermography, have gained attention as potential tools for estrus detection [5]. Changes in peripheral temperature, especially in the vulvar and ocular regions, are associated with increased blood flow and vasodilation driven by endocrine changes during estrus [2,3,4]. Ocular temperature has also been proposed as an indicator of systemic physiological and metabolic status, reflecting stress, inflammation, and thermoregulatory processes [6,7]. The integration of thermal imaging with behavioral data may therefore provide complementary information regarding physiological changes associated with estrus.

Advances in precision livestock farming have also enabled continuous monitoring of internal physiological parameters, such as reticulorumen temperature, through intraruminal sensor technologies. These systems provide continuous real-time physiological data and may contribute to a better understanding of relationships among internal temperature dynamics, behavioral changes, and reproductive events in cattle [8,9,10,11,12,13,14,15,16,17].

Another important factor influencing reproductive performance is nutritional and metabolic status, as metabolic imbalance and oxidative stress during the transition and early postpartum periods may impair ovarian function and delay the resumption of cyclicity [9,10,18]. Plant-derived polyphenols, particularly those found in grape by-products, have attracted interest because of their antioxidant and anti-inflammatory properties and their potential role in supporting metabolic and physiological adaptation during physiologically demanding periods [19,20,21]. Grape extracts contain bioactive compounds such as proanthocyanidins, anthocyanins, flavonoids, and resveratrol, which have been associated with antioxidant activity and modulation of metabolic processes [19,20,21,22]. Previous studies in dairy cattle have suggested possible beneficial effects of grape-derived supplementation on antioxidant status and metabolic adaptation, although findings remain inconsistent and evidence in beef cattle remains limited [19,23,24]. Consequently, further studies under practical production conditions are warranted.

Despite increasing interest in combining physiological monitoring and nutritional interventions, limited information is available on how these approaches interact in the context of postpartum reproductive dynamics. In particular, the relationship between thermal indicators, internal physiological parameters, behavioral expression of estrus, and hormonal profiles under supplementation conditions has not been fully elucidated.

Therefore, the aim of this exploratory pilot study was to evaluate possible associations among thermographic, behavioral, hormonal, and reticulorumen temperature indicators during postpartum reproductive recovery in Angus cows receiving grape extract supplementation. In addition, the study aimed to explore whether supplementation was associated with changes in postpartum reproductive dynamics, including time to first estrus.

## 2. Materials and Methods

All animal-related procedures were carried out in compliance with Directive 2010/63/EU of the European Parliament and of the Council of 22 September 2010 on the protection of animals used for scientific purposes [25]. The study also adhered to the Law of the Republic of Lithuania on Animal Welfare and Protection (Law No. IX-2271) [26], as well as to the relevant implementing regulations issued by the State Food and Veterinary Service of the Republic of Lithuania, which define the requirements for the use, housing, care, handling, and experimental application of animals [27]. The animal study was approved by the Institutional Board of the Animal Science Institute of the Lithuanian University of Health Sciences (protocol No. 24/01/30/01).

### 2.1. Animals and Experimental Design

Nineteen Angus cows (2–10 years of age), including both primiparous and multiparous animals (mean parity: 2.8), were included in the study. Clinical and reproductive examinations were performed within the first 24 h after calving. All cows were clinically healthy, had experienced unassisted calving, and showed no evidence of reproductive disorders or postpartum complications. At the time of enrollment, all cows exhibited a moderate body condition, with body condition scores within the recommended range for beef cattle (3.0–3.5 on a 5-point scale). Multiparous cows were assigned according to parity, whereas primiparous cows (heifers) were randomly allocated. The control group (C) consisted of four primiparous and six multiparous cows (mean parity: 2.8), whereas the treatment group (T) included four primiparous and five multiparous cows (mean parity: 2.9). Animals were divided into two groups: control (C; *n* = 10), without supplementation, and treatment (T; *n* = 9), receiving slow-release grape extract boluses.

All cows were housed in a free-stall facility in Baisogala (Radviliškis district, northern Lithuania). The experimental period lasted from March to June. Artificial insemination was performed between 60 and 70 days postpartum according to the farm reproductive management protocol. From 21 days prior to the expected calving date until 60–70 days postpartum, cows were separated from the main herd and maintained in individual straw-bedded calving pens. All animals were fed a total mixed ration and had free access to water. The diet consisted of 65% grass silage, 25% corn silage, 7% wheat straw, and 3% compound feed (corn, wheat, rapeseed), and was provided once daily.

Cows in the T group received a slow-release bolus containing 14.8 g of dry grape extract (*Vitis vinifera*). A total of five boluses were administered orally every three weeks using a stainless steel balling gun (Ø 34 mm; Bio Enterprise BV, Vroomshoop, The Netherlands) to ensure accurate delivery into the rumen. Supplementation was initiated 21 days before the expected calving date (day −21) and continued at approximately three-week intervals on days 0, 21, 42, and 63 postpartum. Bolus administration was consistently performed between 10:00 and 11:00 a.m. The grape extract was encapsulated within a plant-derived matrix designed to reduce rapid ruminal degradation of bioactive compounds and to allow gradual release of the extract within the reticulorumen over approximately three weeks. The extract was characterized by a high polyphenol content (>60%), including proanthocyanidins (>45%) and anthocyanins (>0.3%).

### 2.2. Sampling Procedures, Hormonal Analysis, and Thermophysiological Measurements

From calving until artificial insemination, blood samples were collected from all cows once weekly at 7-day intervals. Sampling was consistently performed between 10:00 and 11:00 a.m. to minimize the potential influence of circadian rhythms on physiological parameters. Blood samples were aseptically collected from the v. coccygealis into 9 mL vacuum tubes without anticoagulant (VACUTEST, Verona, Italy). Following collection, blood samples were allowed to clot at room temperature for approximately 30–60 min. Samples were then centrifuged at 3000× *g* for 5 min, and the separated serum was transferred into sterile tubes and stored at −60 °C until further analysis.

Serum progesterone and estradiol concentrations were determined at the Animal Reproduction Laboratory of the Veterinary Academy, Lithuanian University of Health Sciences. Hormonal analyses were performed using commercially available ELISA kits for progesterone (RE52231) and 17β-estradiol (RE52041) (IBL International GmbH, Hamburg, Germany), according to the manufacturer’s instructions. The analytical measurement range for both hormones was 0–40 ng/mL. The mean intra-assay coefficient of variation was 6.4%, and the mean inter-assay coefficient of variation was 6.6%. The sensitivity of the assay method was 0.03 ng/mL.

Simultaneously with blood sampling, non-invasive body surface temperature measurements were conducted using infrared thermography. Ocular temperature and vulvar surface temperature were assessed with a portable infrared thermographic camera (FLIR E540, FLIR Systems, Santa Barbara, CA, USA). Ocular and vulvar surface temperatures were assessed using a portable infrared thermographic camera (FLIR E540, FLIR Systems, Santa Barbara, CA, USA) with an accuracy of ±2 °C, thermal sensitivity expressed as noise equivalent temperature difference (NETD) of 0.03 °C, resolution of 464 × 348 pixels, and a temperature measurement range from −20 °C to 400 °C. The emissivity coefficient was set to 0.98, corresponding to the emissivity of animal biological tissues. Ambient temperature and relative humidity inside the barn were monitored during thermographic assessments using a ThermoPro TP50 digital thermohygrometer (ThermoPro, Shenzhen, China) and were incorporated into the FLIR ResearchIR software settings during image analysis to minimize the influence of environmental variation on thermal measurements. During the study period, ambient temperature ranged from 18 to 22 °C and relative humidity from 40% to 63%. Thermal images were acquired at a standardized distance of approximately 1 m under controlled indoor environmental conditions, avoiding direct sunlight, wind, and moisture to ensure measurement accuracy and repeatability.

**Figure 1:**
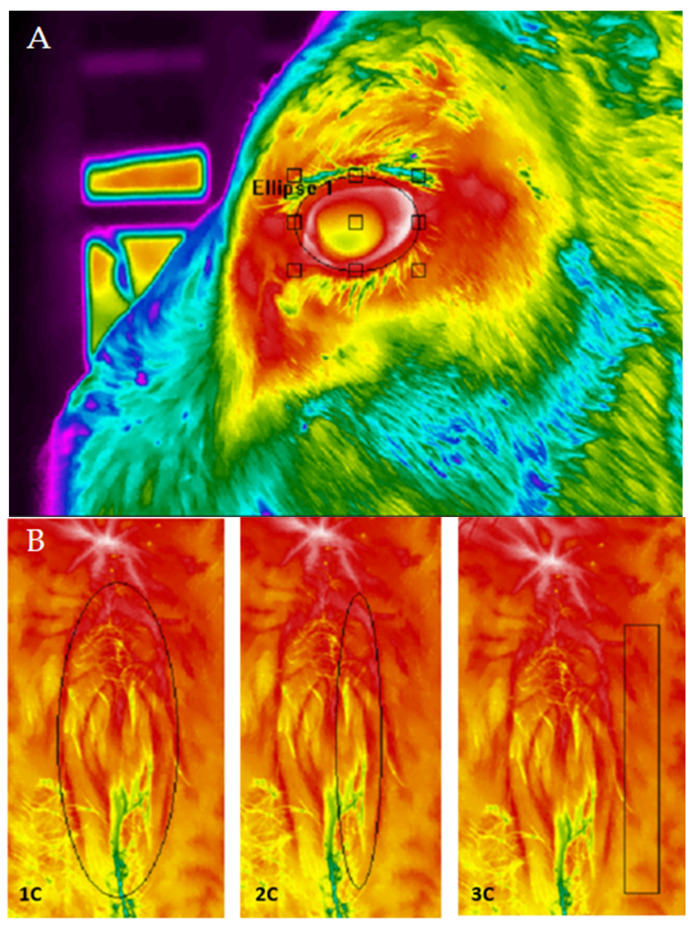
Thermographic images and measurement areas were used for temperature assessment in the examined regions. (**A**): ocular region; (**B**): vulvar region; 1C—entire vulva; 2C—central elongated area of the vulva; 3C—surrounding area of the vulva.

Thermographic images were recorded and analyzed using a rainbow color palette, where lower temperatures were represented by dark blue and higher temperatures by white, with intermediate colors indicating transitional values. Image analysis was performed using dedicated software (FLIR ResearchIR, version 4.40; Teledyne FLIR, Wilsonville, OR, USA). For each region of interest (ROI), mean, minimum, and maximum surface temperatures were calculated using FLIR ResearchIR software. Three thermographic images were obtained for each anatomical region during each assessment session, and the image with the best anatomical positioning and focus was selected for analysis. Regions of interest were manually selected based on consistent anatomical landmarks for each imaging region. Although slight variations in animal posture, image angle, and positioning could occur during image acquisition, all thermographic assessments were performed by the same operator using standardized camera distance, acquisition angle, environmental conditions, and software settings to minimize variability among measurements. Because the exact ROI size and pixel number could vary slightly between images, thermographic evaluation was based on temperature measurements obtained from predefined anatomical regions rather than analysis of individual pixel intensity patterns. For statistical analysis, maximum surface temperature was selected because this parameter demonstrated the lowest dispersion and highest repeatability among thermographic measurements (Figure 1).

### 2.3. Reticulorumen Temperature Monitoring

Reticulorumen temperature (RT) was continuously monitored throughout the entire study period using intraruminal temperature-sensing boluses (smaXtec animal care technology^®^, Graz, Austria). The boluses had been administered to the grazing herd approximately one year before the beginning of the study and remained in place throughout the experimental period. The boluses were administered orally into the reticulorumen following the manufacturer’s guidelines, using a dedicated applicator. Each cow received a single bolus, which was administered by the same experienced veterinarian to ensure procedural consistency. During administration, cows were restrained in a self-locking stand. The head was manually stabilized, the mouth was opened, and the bolus was placed at the base of the tongue to allow voluntary swallowing. Due to their density and design, the boluses settled naturally in the reticulum. After administration, cows were observed for approximately two hours to ensure that no regurgitation of the bolus occurred.

Prior to administration, each bolus was activated, assigned to the individual cow by linking it to the corresponding ear tag, and connected to the central monitoring system. Reticulorumen temperature data were automatically recorded at 10 min intervals and transmitted wirelessly via antennas to the central database. Data acquisition, storage, and preliminary processing were managed using smaXtec messenger^®^ software (version 4).

### 2.4. Evaluation of Infrared Thermography Reliability and Reproductive Responses

To explore associations among thermographic and physiological indicators, surface temperature data obtained from ocular and vulvar regions were compared with reticulorumen temperature measurements described in Section 2.3.

Temperature values were synchronized with the time points of thermographic image acquisition. Corresponding reticulorumen temperature values were extracted and analyzed in relation to ocular and vulvar surface temperatures to assess potential relationships between internal and peripheral temperature measurements during the postpartum period.

### 2.5. Assessment of First Postpartum Estrus

The first postpartum estrus was determined in cows from both the C and T groups based on reticulorumen temperature data recorded by the smaXtec system. Estrus-related behavioral signs observed during routine weekly examinations were recorded as supportive clinical observations. The interval from calving to the first detected estrus was calculated for each cow. Thermographic temperature changes observed during the first postpartum estrus were analyzed and compared with reticulorumen temperature data, allowing the assessment of physiological responses associated with estrus expression.

Time to first postpartum estrus was also analyzed using Kaplan–Meier survival analysis, with estrus occurrence defined as the event. Differences between groups were evaluated using the log-rank (Mantel–Cox) test. Survival curves were generated to illustrate the probability of remaining anestrous postpartum over time.

### 2.6. Conception Rate

Conception rate was calculated as the ratio of the number of pregnant cows to the number of inseminated cows and expressed as a percentage, according to Qureshi et al. (2008) [28].

$$\mathrm{Conception}\;\mathrm{rate}=\frac{n\;\mathrm{pregnant}\;\mathrm{cows}}{n\;\mathrm{inseminated}\;\mathrm{cows}}\times 100$$

### 2.7. Statistical Analysis

All statistical analyses were performed using IBM SPSS Statistics (version 30.0; IBM Corp., Armonk, NY, USA) and R software (version 4.4.1; R Foundation for Statistical Computing, Vienna, Austria).

Pearson correlation coefficients (r) were calculated to evaluate relationships among thermal, physiological, hormonal, and behavioral parameters. Analyses were conducted separately for C and T groups and for two analytical periods: the full study period and the first estrus period. Pairwise correlations were calculated using pairwise complete observations. Correlation matrices included correlation coefficients, two-tailed *p*-values, and sample sizes (N).

To control for multiple comparisons, *p*-values were adjusted using the Benjamini–Hochberg false discovery rate (FDR) procedure, applied separately within each correlation matrix. Adjusted *p*-values are reported as q-values. Correlations were considered statistically significant at q < 0.05. Correlation strength was interpreted as follows: weak (|r| < 0.30), weak–moderate (0.30–0.49), moderate (0.50–0.69), and strong (≥0.70). Due to the limited sample size in the first estrus dataset, results from this period were interpreted with caution, with emphasis on effect sizes.

Repeated measurements obtained during estrus and non-estrus periods were analyzed using linear mixed-effects models (LMM). The general model structure was:

$$Y_{ijk}=\mu +G_{i}+P_{j}+(G\times P{)}_{ij}+Cow_{k}+\varepsilon_{ijk}$$

where *Y* represents the dependent variable, μ is the overall mean, *G* is the fixed effect of group (C or T), *P* is the fixed effect of reproductive period (estrus or non-estrus), (*G* × *P*) is the interaction between group and reproductive period, Cow is the random effect of individual animal, and *ε* is the residual error.

Models were fitted using restricted maximum likelihood (REML), and denominator degrees of freedom were approximated using the Satterthwaite method. Fixed effects were evaluated using Type III tests.

When mixed models were not suitable due to convergence issues or data structure limitations, a change-score approach was applied. Differences between periods were calculated as:

$$\Delta Y=Y_{non-estrus}-Y_{estrus}$$

These values were subsequently analyzed using a general linear model (GLM):

$$\Delta Y_{ik}=\mu +G_{i}+\varepsilon_{ik}$$

where *G* represents the fixed effect of the group. Type III tests were used, and the effect size was reported as partial eta squared (partial η^2^).

Statistical significance was set at *p* < 0.05, while 0.05 ≤ *p* < 0.10 was considered a tendency. Results are presented as least-squares means ± standard error (SE), unless otherwise indicated.

The estrus index was generated using a commercial cloud-based artificial intelligence (AI) system (smaXtec animal care technology^®^, Graz, Austria) integrating behavioral and physiological sensor data with proprietary adjustment factors (e.g., production-related and temporal parameters). As this index represents a composite digital indicator rather than a direct biological measurement, results involving this variable were interpreted accordingly.

## 3. Results

### 3.1. Kaplan–Meier Analysis of Time to First Postpartum Estrus

A significant effect of supplementation was observed for the change in days to estrus between reproductive periods (F_1.17_ = 4.56; *p* = 0.048; partial η^2^ = 0.21) (Table 1).

**Figure 2:**
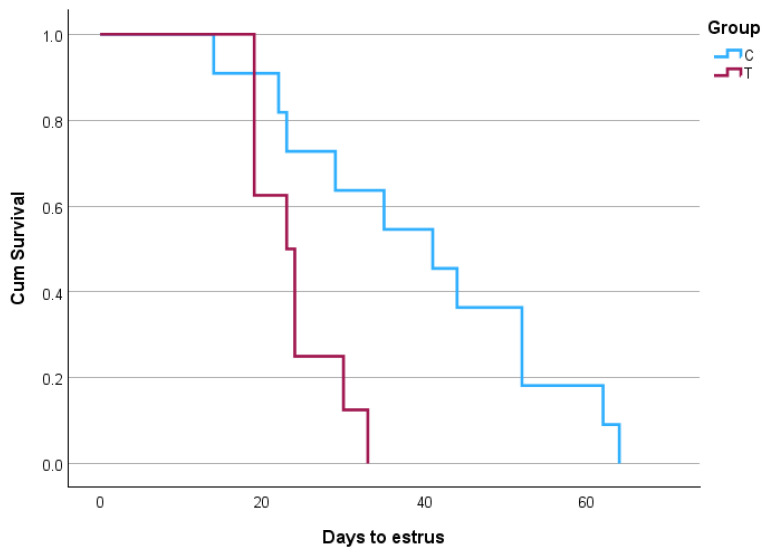
Kaplan–Meier survival curves for time to first postpartum estrus in C and T groups.

Kaplan–Meier survival analysis demonstrated significant differences in time to first postpartum estrus between the C and T groups (Figure 2). The probability of remaining anestrous postpartum decreased more rapidly in the T group compared to the C group. Differences between groups were evaluated using the log-rank (Mantel–Cox) test.

Cows in the T group returned to estrus earlier postpartum compared to the C group. Mean time to first estrus was 23.88 ± 1.86 days in the T group and 39.82 ± 5.05 days in the C group. Median time to first estrus was 23.0 days (95% CI: 18.38–27.62) in the T group and 41.0 days (95% CI: 24.82–57.18) in the C group.

The log-rank (Mantel–Cox) test indicated a significant difference between survival distributions (χ^2^ = 6.642, df = 1, *p* = 0.010), confirming an earlier onset of postpartum estrus in supplemented cows.

### 3.2. Effects of Supplementation and Reproductive Period

No significant effects of supplementation were observed for reticulorumen temperature, activity, ocular temperature, vulvar temperature, estradiol, or progesterone concentrations (*p* > 0.05).

A tendency toward a group × reproductive period interaction was observed for the estrus index (*p* = 0.050).

### 3.3. Conception Rate

**Figure 3:**
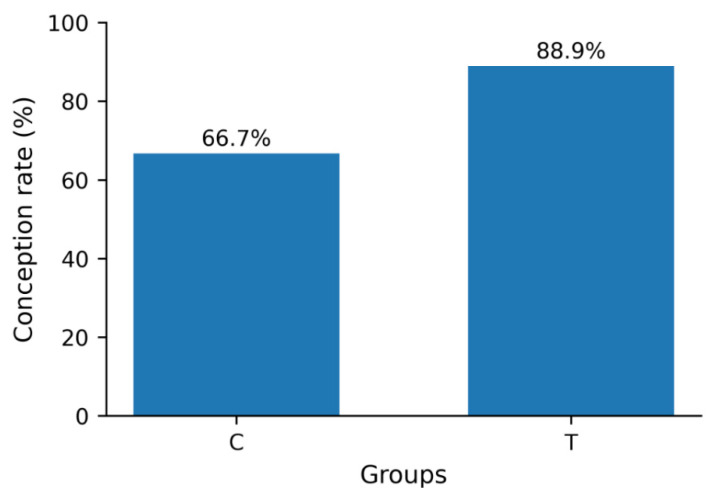
Comparison of conception rate between experimental groups.

The conception rate was numerically higher in the T group (88.9%) compared with the C group (66.7%) (Figure 3). However, due to the limited sample size, this difference was not statistically significant.

### 3.4. Correlation Analysis

Following Benjamini–Hochberg false discovery rate correction, only significant correlations (q < 0.05) were retained (Table 2). Moderate associations were identified between vulvar and ocular temperatures.

Reticulorumen temperature was positively associated with activity and estrus index in both groups. Activity and estrus index were also moderately correlated in both groups.

Overall, the correlation analysis was exploratory and aimed to characterize relationships among physiological and behavioral indicators rather than validate diagnostic performance.

## 4. Discussion

The principal finding of this pilot study was the significantly shorter interval to first postpartum estrus in cows receiving grape extract supplementation (23.88 ± 1.86 days) compared with control cows (39.82 ± 5.05 days). Although most physiological, thermographic, and hormonal variables did not differ significantly between groups, the earlier return to estrus suggests a potential improvement in postpartum reproductive recovery. This finding may have practical relevance for extensively or semi-extensively managed beef herds, where prolonged postpartum anestrus is a major factor limiting reproductive efficiency [29,30]. A shorter interval to first estrus could increase the proportion of cows conceiving within a restricted breeding season and contribute to improved herd reproductive performance. However, given the exploratory nature of the study, the limited sample size, and the absence of measurements related to oxidative stress, inflammation, metabolism, or energy balance, the biological mechanisms underlying this response remain unclear and require further investigation.

Previous studies have demonstrated that postpartum reproductive recovery is closely associated with energy balance, metabolic adaptation, and overall physiological status [18,19,22,23,24]. Plant-derived polyphenols have attracted interest due to their antioxidant and anti-inflammatory properties and their potential role in supporting physiological adaptation during metabolically demanding periods [21,31,32,33]. During the transition and early postpartum periods, beef cows experience increased production of reactive oxygen species (ROS), which may impair ovarian follicular development and delay the resumption of cyclicity [8]. Therefore, the polyphenol-rich grape extract used in this study (>60% polyphenols), containing bioactive compounds such as proanthocyanidins and anthocyanins, may have contributed to improved postpartum ovarian activity. However, oxidative stress, inflammatory, metabolic, and energy balance indicators were not evaluated in the present study. Consequently, the mechanisms underlying the earlier onset of estrus in supplemented cows remain unclear and warrant further investigation.

Exploratory associations were observed among thermographic, behavioral, and reticulorumen temperature indicators in both groups. Moderate relationships between vulvar and ocular temperatures may indicate that peripheral thermal changes are associated with physiological responses occurring during estrus. Previous studies have similarly suggested that infrared thermography may provide complementary physiological information related to reproductive status in cattle [2,3,4,11]. However, because diagnostic accuracy was not evaluated in the present study, these findings should not be interpreted as validation of estrus detection performance.

Behavioral activity and the AI-derived estrus index were also associated with reticulorumen temperature in both groups. These findings are consistent with previous reports indicating that activity-related variables remain among the most informative indicators associated with estrus expression in cattle [8,9,13,14,16,20]. Nevertheless, the estrus index used in the present study was generated using a proprietary commercial algorithm integrating multiple sensor-derived inputs. Consequently, its biological interpretation remains limited, and the observed associations should be considered exploratory.

The conception rate was numerically higher in supplemented cows (88.9% in the T group and 66.7% in the C group); however, because of the small sample size, this observation should be interpreted descriptively without inference regarding reproductive efficiency. Similarly, several correlations identified during the first estrus period did not remain significant after correction for multiple comparisons, most likely reflecting limited statistical power.

Overall, the present findings suggest that multimodal physiological monitoring may provide complementary information associated with postpartum estrus expression in beef cows. However, the study was exploratory in nature and was not designed to validate estrus detection systems or diagnostic performance. Additional studies involving larger populations, more frequent endocrine assessment, evaluation of metabolic and oxidative biomarkers, and validation of diagnostic accuracy are required before practical conclusions regarding estrus monitoring approaches can be established.

## 5. Conclusions

This exploratory pilot study demonstrated preliminary associations among thermographic, behavioral, and reticulorumen temperature indicators during postpartum estrus in beef cows. Grape extract supplementation was associated with an earlier onset of postpartum estrus, although most individual physiological and hormonal variables were not significantly affected. The findings suggest that multimodal physiological monitoring may provide complementary insights into estrus; however, due to the exploratory study design and limited sample size, further studies are required to validate these preliminary observations and clarify the potential role of grape-derived polyphenols in postpartum reproductive dynamics.

## Figures and Tables

**Table 1:** Effects of grape extract bolus supplementation and reproductive period on physiological and reproductive parameters.

Parameter	Model	Group (*p*)	Interaction (*p*)	Effect Size (η^2^)
Days to estrus	GLM	0.048	—	0.21
Estradiol	LMM	0.437	0.767	—
Progesterone	GLM	0.893	—	—
Estrus index	LMM	0.117	0.050	—
Mean temperature (7 d)	GLM	0.531	—	—
Activity	GLM	0.367	—	—
Reticulorumen temp	LMM	0.231	0.362	—
Eye temperature	GLM	0.685	—	—
Vulvar temp 3C	GLM	0.176	—	0.147
Vulvar temp 2C	LMM	0.498	0.522	—
Vulvar temp 1C	LMM	0.673	0.917	—

Note: LMM—linear mixed model; GLM—generalized linear model; η^2^—partial eta squared; *p* < 0.05 considered statistically significant.

**Table 2:** Exploratory correlations among selected physiological and behavioral indicators after FDR correction (q < 0.05).

Variables	Control (r)	q-Value	Treatment (r)	q-Value
Vulvar temp—Ocular temp	0.37–0.50	<0.05	0.43–0.52	<0.05
Reticulorumen temp—Activity	0.72	<0.001	0.48	<0.05
Reticulorumen temp—Estrus index	0.75	<0.001	0.60	<0.05
Activity—Estrus index	0.65	<0.001	0.66	<0.001

Note: q-values adjusted using the Benjamini–Hochberg method.

## Data Availability

The data presented in this study are available on request from the corresponding author.
